# Beyond the mono-nucleosome

**DOI:** 10.1042/BST20230721

**Published:** 2025-01-31

**Authors:** Juliana Kikumoto Dias, Sheena D’Arcy

**Affiliations:** Department of Chemistry and Biochemistry, The University of Texas at Dallas, Richardson, Texas, 75080, USA

**Keywords:** chromatin, multi-nucleosome array, chromatin remodeler, nucleosome

## Abstract

Nucleosomes, the building block of chromatin, are responsible for regulating access to the DNA sequence. This control is critical for essential cellular processes, including transcription and DNA replication and repair. Studying chromatin can be challenging both *in vitro* and *in vivo*, leading many to use a mono-nucleosome system to answer fundamental questions relating to chromatin regulators and binding partners. However, the mono-nucleosome fails to capture essential features of the chromatin structure, such as higher-order chromatin folding, local nucleosome–nucleosome interactions, and linker DNA trajectory and flexibility. We briefly review significant discoveries enabled by the mono-nucleosome and emphasize the need to go beyond this model system *in vitro*. Di-, tri-, and tetra-nucleosome arrays can answer important questions about chromatin folding, function, and dynamics. These multi-nucleosome arrays have highlighted the effects of varying linker DNA lengths, binding partners, and histone post-translational modifications in a more chromatin-like environment. We identify various chromatin regulatory mechanisms yet to be explored with multi-nucleosome arrays. Combined with in-solution biophysical techniques, studies of minimal multi-nucleosome chromatin models are feasible.

## Introduction

In eukaryotes, genomic DNA is packaged into chromatin, with nucleosomes being the dynamic foundational unit. Nucleosomes comprise ~147 bp of double-stranded DNA that wraps around a histone protein octamer ~1.7 times [1]. The histone octamer consists of two copies of each canonical histone: H2A, H2B, H3, and H4. The linker histone H1 binds to the nucleosome at the entry and exit of the DNA and causes chromatin to adopt a compacted state [2,3]. Adjacent nucleosomes connect via linker DNA, the length of which depends on species and genomic location [4]. The linker DNA length substantially impacts higher-order chromatin folding patterns [5,6]. The N-terminal tails of all four core histones and the C-terminal tail of H2A are highly disordered and are targets for post-translational modification (PTM), which can modulate nucleosome interactions. The histone tails provide binding platforms for various transcriptional and DNA maintenance machinery. Histone tails can interact with nucleosomal and linker DNA, contributing to chromatin folding [7,8]. The combination of histones, nucleosomal DNA, and linker DNA interactions dictate higher-order chromatin compaction [4,7-10]. Since the first crystal structure of the mono-nucleosome almost 30 years ago, chromatin researchers have answered many questions regarding mechanisms of chromatin regulation, including histone chaperoning [11], nucleosome binding (Table 1), nucleosome remodeling [29,30], and histone PTM [31-35]. However, higher-order chromatin folding, nucleosome dynamics, and some mechanisms of chromatin remodeling remain underexplored due to the confines of the frequently used mono-nucleosome model system. There is an increasing need to go beyond the mono-nucleosome to use multi-nucleosome arrays that more closely mimic the *in vivo* chromatin environment.

**Table 1:** Solved crystal structures of mono-nucleosome in complex with a binding partner from 2021 to 2023.

Binding partner	Relevance	Chromatin-related effects	Citation
ALC1	Loss of ALC1 results in lethality of recombinant-deficient cells	Nucleosome sliding	[12,13]
Chd1	Gene activation	Unwrapping of terminal DNA	[14]
Chd4	Involved in gene repression. Mutations are associated with human cancer or intellectual disability disorder	DNA distortion at SHL + 2	[15]
DDM1	Transcription silencing Heterochromatin remodeler for methyl transferase access	Nucleosome compaction and shorter linker length preference	[16]
INO80	Binds both nucleosomes and hexasomes, at opposite orientations, with a preference for hexasome	Possible hexasome formation, and hexasome and nucleosome sliding	[17,18]
SWI/SNF subcomplex, RSC, and FH	High occurrence of cancer in mutations	DNA unwrapping and histone ejection from DNA	[19,20]
PBAF	Subunit mutations are associated with human cancer and intellectual disability syndromes	Histone tail recognition sites Create nucleosome-depleted regions	[21]
Chd1/FACT	Transcription initiation complex	DNA unwrapping, nucleosome sliding, and transcription patterns	[22]
SETD2	Complex inhibited by oncogenic mutations	DNA unwrapping, longer linker length preference Binds at the n-terminal of H3 and SHL 1	[23]
SUV420H1	Overexpression initiates a cascade of events related to human cancer	H4K20 methylation on H2A.Z nucleosome. Binds the H4 tail and acidic patch	[24]
Rpd3S	Works with H1 to regulate gene silencing	Nucleosome deacetylationInteracts and reconfigures linker DNA	[25]
CBF3	Regulates chromosome segregation by depositing centromeric protein A (CENPA) variant	The complex binds to linker DNA and core histones in a sequence-dependent manner	[26]
TBP	Assembles and transcribes a transcription complex close to gene promoters	TBP binds to the W601 nucleosome core particle (NCP) at the TATA sequence toward the end of nucleosomal DNA and at the GC motif at the dyad	[27]
OCT4	Induces cell reprogramming by targeting closed chromatin	Multiple units of OCT4, unwrap ~25 bp, even in the H1 bound NCP	[28]

*In vitro*, several factors dictate higher-order chromatin compaction, including ionic strength [36], DNA sequence and length [37], histone tail PTMs [38], and inter-nucleosome interactions [8]. The chromatin structure begins as a string of nucleosomes with no compaction pattern [39], and at low ionic strength, local interactions between nucleosomes induce chromatin to fold into a 30 nm fiber [39]. This structure can present itself as a two-start (zigzag) or one-start (solenoid) fold [40,41]. The central difference between both structures is one or two nucleosome stacking patterns, with the one-start being more dense. Physiological or higher ionic strength neutralizes the negative charges from the DNA, leading to a condensate with no repetitive pattern of interactions between nucleosomes [42]. Inter-nucleosome interactions are multifactorial and include nucleosome–nucleosome and histone-linker DNA. Nucleosome arrays indicated that the length of linker DNA and its sequence can affect higher-order chromatin folding [8]. Shorter DNA linkers increased nucleosome stacking stiffness, while longer lengths presented a more flexible nucleosome–nucleosome stacking conformation [8]. Arrays also showcase the histone–linker DNA interactions, where the deletion of the H4 histone tail prevents nucleosome stacking in long linker-length chromatin fibers [8]. Still, it did not affect the folding of shorter linker-length fibers [7,43], likely due to other protein–protein interactions. Notably, the H3 histone tails also interact with nucleosomal DNA and linker DNA at the exit and entry points, suggesting that the H3 tails contribute to chromatin compaction [44,45]. These results highlight that the inter-nucleosome interactions, such as the dynamic contact points, are multifactorial in dictating chromatin folding patterns.

A widely used tool to study nucleosomes *in vitro* is DNA positioning sequences. For example, the Widom 601 (W601) has a high binding affinity to the histone octamer and can assemble stable nucleosomes [46]. The W601 contains 282 bp, of which 147 bp wrap around the histone octamer, and the remaining 137 bp make up symmetrical DNA linkers on both sides of the nucleosome. The sequence features a ten-base pair repeat of TA nucleotide dimers [46] and this repetitive sequence facilitates DNA bending around the histone octamer. Positioning sequence models are crucial in making high-resolution structural work possible due to higher nucleosome stability [15,21,47-49] although the W601 does have a similar unwrapping energy to native yeast nucleosomes [50]. The high binding affinity of DNA to histones could result in resistance to changes upon binding partner incorporation or nucleosome modifications. Positioning sequences enable studies of multi-nucleosome arrays, especially high-resolution structural approaches that reveal DNA trajectories and nucleosome–nucleosome interactions. However, there is a possibility that the high positioning limits the ability to detect inter-nucleosome dynamics.

### Nucleosome arrays for the analysis of regulatory mechanisms

Nucleosome–nucleosome interactions play an essential role in chromatin compaction. The modification of one nucleosome can directly or indirectly affect its adjacent neighbors [51,52]. Increasing linker length causes twisting of the chromatin structure [53], resulting in an inward or outward chromatin conformation. The binding of chromatin remodelers to modify nucleosome structure and composition can indirectly cause neighboring nucleosomes to compact, loosen, or slide along DNA [12,14,54]. The epigenetic marker CENPA and histone variant H2A.B increase flexibility at the entry and exit site of DNA [49,55], causing twisting and conformational changes in adjacent nucleosomes. Nucleosome arrays are constructed in two ways: (1) by ligating multiple nucleosomes together or (2) by utilizing a longer strand of histone-binding DNA, in which multiple nucleosomes will form on the same DNA. The first has the advantage of controlling nucleosome positioning and composition, while the second allows for arrays with more nucleosomes.

**Figure 1:**
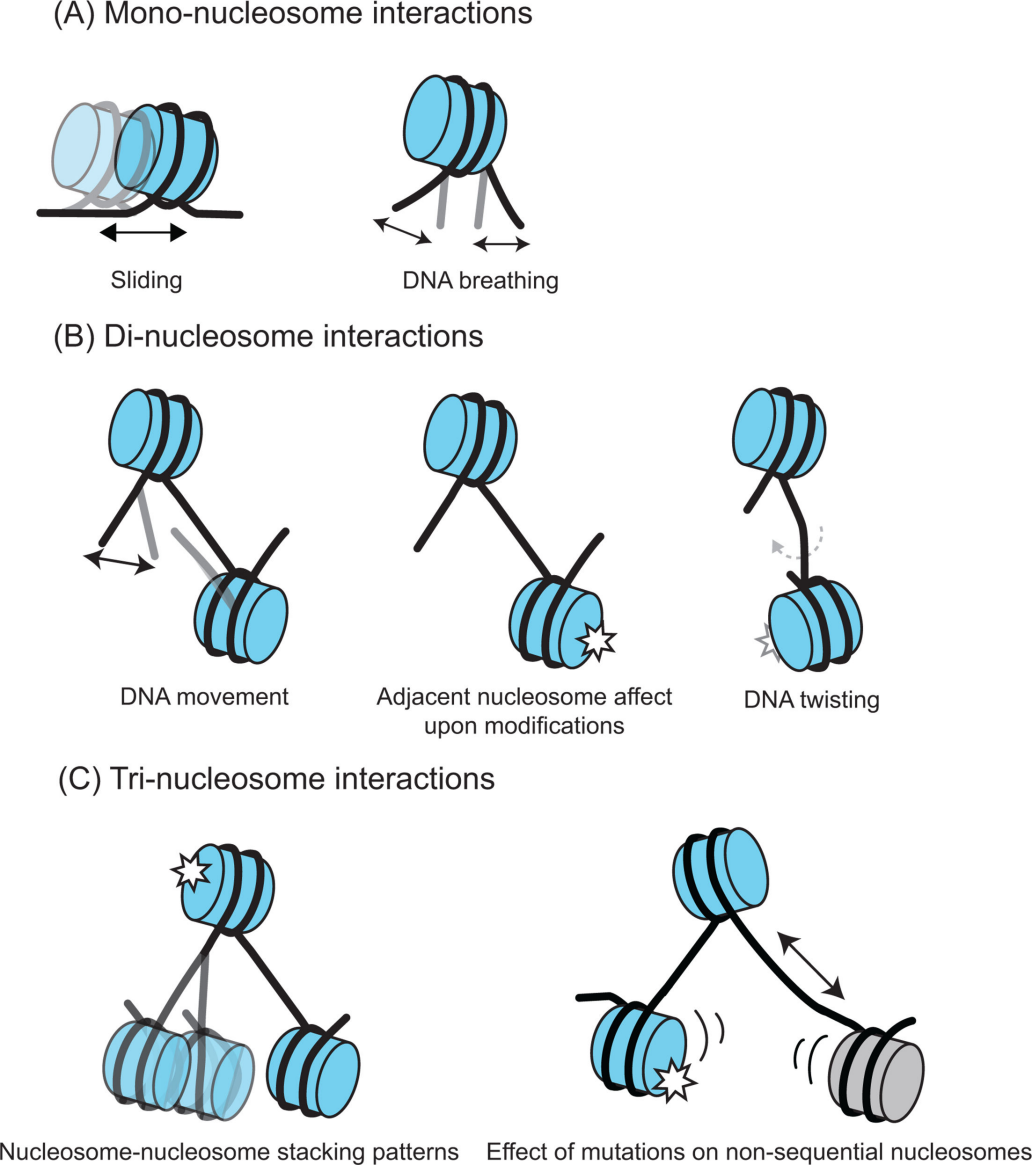
Chromatin qualities visualized by minimal array systems. (**A**) Mono-nucleosome systems show alterations in histone–histone contacts within the octamer and histone–DNA interfaces within a nucleosome. (**B**) Di-nucleosomes additionally show DNA trajectory between NCPs and effects on adjacent nucleosome upon modifications (white star) such as PTMs and variant incorpoaration. (**C**) Tri-nucleosomes have the added benefit of demonstrating nucleosome–nucleosome stacking and effects on non-sequential nucleosomes.

Nucleosome arrays can vary in length from di-nucleosomes to 30-mer arrays (see [56-58] for longer arrays). For feasibility purposes, di-, tri-, and tetra-nucleosome arrays have been used more frequently (Figure 1) and adopt various conformations. Nucleosomes 1 and 2 of tri-nucleosome arrays appeared to be stacked, suggesting that di-nucleosomes are the minimal folding unit [59]. The tri-nucleosome arrays also showed stacking of nucleosomes 1 and 3, supporting the theory that the tri-nucleosome could be the minimal structural unit [60]. Li et al. [61] showed that a chromatin fiber with H5 linker histone bound revealed a helical structure resembling the two-start fold, with tetra-nucleosome as the minimal structural unit.

**Figure 2:**
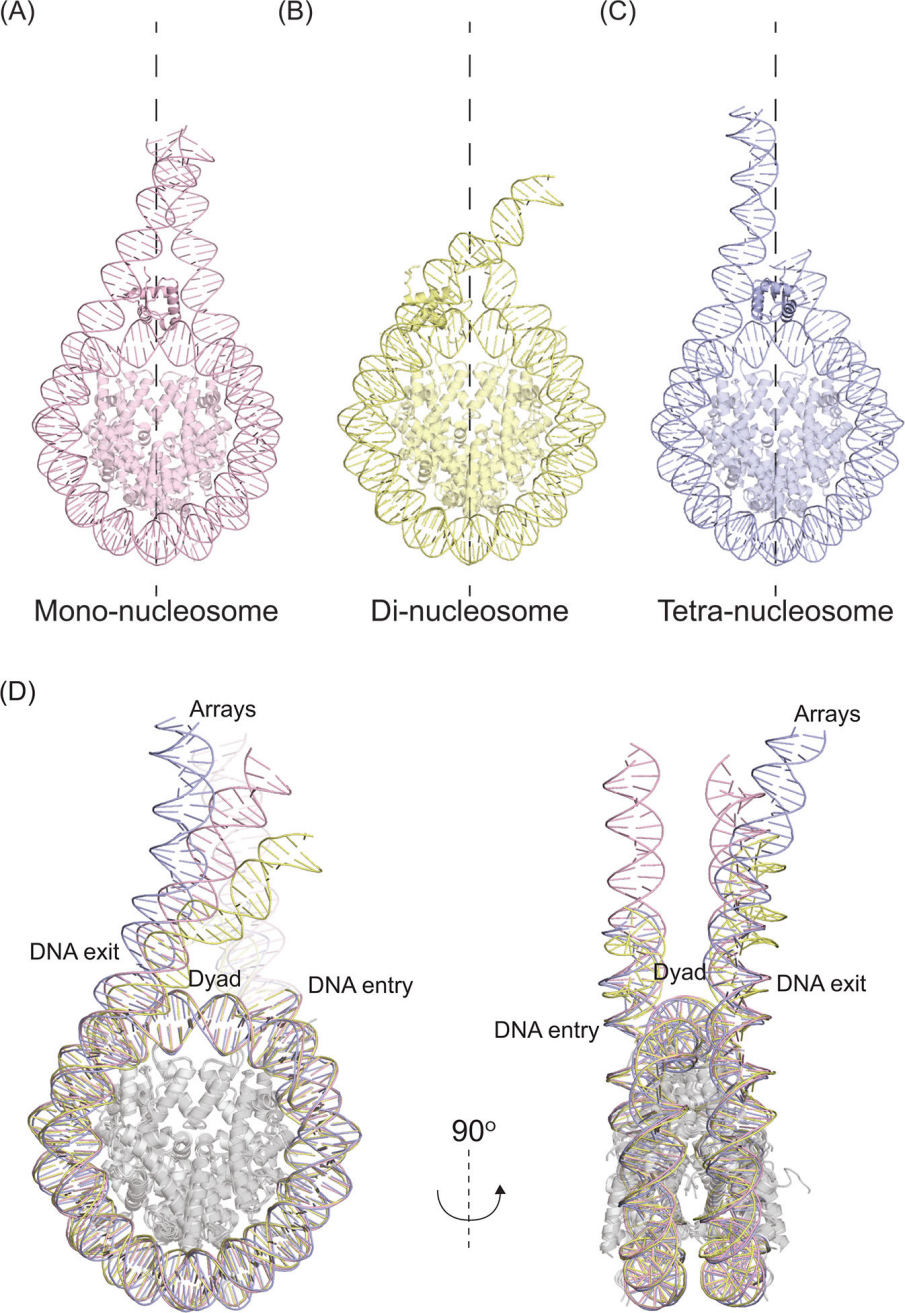
NCP1 of minimal nucleosome arrays compared to the mono-nucleosome each with linker histone H1. (**A**) Solved structure of a 197 bp nucleosome in complex with linker histone H1.4 (PDB:7K5Y). (**B**) NCP1 of a 338 bp di-nucleosome bound to linker histone H1.X (PDB:6L9Z). (**C**) NCP1 of the 4×177 bp nucleosome array containing H1.4 (PDB:7PET). (**D**) The superposition of structures A–C, with respect to (**H3-H4**) _2_, highlights the DNA orientation difference at the entry and exit points of DNA.

When analyzing regulatory mechanisms in nucleosome arrays, changes in the DNA pathway, histone–DNA interactions, and nucleosome disruptions will be more apparent when these modifications happen to adjacent or non-sequential nucleosomes (Figure 1). The currently solved structures of multi-nucleosome arrays (summarized in Table 2) reveal changes not previously seen in the adjacent nucleosomes. Di-nucleosome arrays have been solved, revealing important nucleosome formation intermediates [66]. The structures have been valuable in assessing DNA twisting between nucleosomes and possible allosteric effects upon binding of the switch/sucrose non-fermentable (SWI/SNF) complex [19]. The SWI/SNF remodeler is known for disassembling nucleosomes and making DNA available for transcription and repair [19]. The di-nucleosome structure acted as a model system to elucidate the remodeler’s mechanism of action [66]. Based on biochemical assays, the authors postulate that two nucleosomes ‘bump’ into each other upon the addition of SWI/SNF. This releases one copy of H2A-H2B, creating one hexasome, and retaining a second canonical nucleosome. Without the array, the nucleosome appears unmodified upon binding to SWI/SNF, highlighting that the use of multi-nucleosome arrays was pivotal for observing remodeling mechanisms [19]. Another example is the di-nucleosome structures with and without linker histone H1. These answered important mechanistic questions about H1 binding sites and the compaction mechanism of chromatin in an H1-bound state (Figure 2) [62,63]. For instance, the di-nucleosome array with single-stranded overhang ends simulates how DNA wraps to accommodate multiple nucleosomes [62]. Different isoforms of H1 have exhibited different flanking DNA conformations [69], but using a di-nucleosome model could extend this analysis to observe its effects on chromatin folding. Mono-nucleosomes with cohesive-ended DNA termini self-assembled with other complementary DNA termini from nearby NCPs but did not show any changes in nucleosome dynamics [70].

**Table 2:** Summary table of currently solved crystal and cryo-EM structures of nucleosome arrays.

	Array	Description	Significance	Citation
*Histone variants*	Di	H1 Crystallography	Library of DNA constructs with high octamer binding affinity for array assembly	[62]
	Di	Cohesive-ended DNA H1 Crystallography	Zigzag conformation DNA linker showed extreme bending	[63]
	Tri	CENPA Cryo-EM	CENPA array showed twisting conformation Canonical array showed no DNA twisting	[53]
	Tetra	H1 Crystallography	First nucleosome array *in vitro* Array shows zigzag conformation	[64]
	Hexa	H1 low ionic strength Crystallography	Zigzag conformation with lower packing density compared to the 30 nm fiber	[65]
*Remodelers*	Di	Remodeling intermediate Crystallography	Nucleosome intermediate formation upon remodeler binding	[66]
	Di	Imitation switch (ISWI) Cryo-EM	Two different subunits of the remodeler recognize NCP1 and NCP2	[67]
*Linker length*	Tri	Linker length: 22 bp and 30 bp, MgCl_2_ Cryo-EM	The presence of MgCl_2_ and longer linker length showed DNA twisting	[53]
	Tri	Linker length: 30 bp, 40 bp, 50 bp, and 60 bp Cryo-EM	Array stability decreases with increasing linker length H1 has a higher binding affinity in longer linker lengths	[60]
	Tetra	11 bp linker length Crystallography	Three different folding conformations show heterogeneity	[68]
*Telomeric DNA*	Di	Arrays reconstituted in telomeric DNA Cryo-EM	Telomeric structures displayed close stacking of nucleosomes Structures show unusually short NRLs	[59]
	Tetra	Arrays reconstituted in telomeric DNA Cryo-EM	Telomeric structures displayed close stacking of nucleosomes Structures show unusually short NRLs	[59]

NRL, nucleosome repeat length.

One disadvantage of di-nucleosomes is their symmetry, making distinguishing between nucleosomes 1 and 2 challenging. One way to overcome this is to identify one with a fluorescent label [71]. Tri-nucleosome arrays, in contrast, have the asymmetry advantage as the flanking nucleosomes are distinguishable from the middle one. These arrays show specific chromatin changes upon histone variant incorporation and linker length variations [53]. Tri-nucleosomes with centromeric histone variant CENPA have been assembled [53]. The authors found that incorporated CENPA leads to DNA twisting, and tri-nucleosome structures had significant DNA bending (curve over a few base pairs, causing a change in orientation) in their folding conformation [53]. In addition to variant incorporation, tri-nucleosomes show the effects of MgCl_2_ concentration and linker length [72]. The structure of the tri-nucleosome was more compact in the presence of higher concentrations of MgCl_2_ and more flexible as the linker length increased. The H1-bound tri-nucleosome also shows that the H1 binding position depends on linker length [60], meaning that H1 needs nucleosomes closer together to compact chromatin properly. Tri-nucleosome arrays are a valuable model, and combined with non-static, in-solution techniques, they can provide robust chromatin analysis. Contrast variation small-angle x-ray scattering (SAXS) was used to analyze arrays with 60 bp linkers [73]. This technique is valuable when analyzing higher-order chromatin because it does not involve labeling and allows tracking of the DNA pathway without interference from protein signals [73]. The tri-nucleosomes in solution showed two distinct populations, one with the flanking nucleosomes stacked together and one where the flanking nucleosomes showed no interaction [73]. This pattern extends to a more physiologically relevant condition with stabilizing divalent ions [53].

Longer arrays, such as the tetra-nucleosomes, provide another versatile model for chromatin folding patterns. Tetra-nucleosomes have been reconstituted using a telomeric-specific DNA sequence [59]. These arrays are present in two states: a string of nucleosomes under low salt or a columnar formation under low Mg^2+^ concentration. The nucleosome arrays formed on a 4×157 bp W601 sequence exhibited more stability and less mobility than the telomeric DNA sequence. Most of the 4×1 57 bp telomeric sequence tetra-nucleosomes were in the columnar conformation, while the W601 tetra-nucleosome was primarily in the zigzag conformation [59]. The tetra-nucleosome model also demonstrated that the columnar conformation displays a shorter than usual nucleosome repeat length (NRL) of ~132 bp, indicating tighter histone-DNA contacts [59]. Despite showing lower stability compared to W601 NCP, the telomeric mono-nucleosome did not provide information on higher-order chromatin folding of telomeric regions [74]. The nucleosome–nucleosome interactions that define the previously observed folding motifs are apparent from the cryo-EM density maps. H2A–H2A′ interactions through the C-terminal domain (nucleosome 1) and loop 1 (nucleosome 2) bridged the nucleosomes. The N-terminal tail of H3 was proximal to the minor grove between nucleosomes 1 and 2, suggesting that H3 can act as a DNA anchor point [59]. This exemplifies the dependence of specific DNA sequences for observing chromatin mobility. These examples showcase the importance of inter-nucleosome interactions and how arrays have helped the field utilize *in vitro* techniques better to understand the higher-order folding patterns of chromatin fibers.

Four different tetra-nucleosomes were assembled using different NRLs [60]. The four NRLs are representative lengths found in active promotor regions (177 bp), gene bodies (186 bp and 197 bp), and heterochromatin (207 bp). It was hypothesized that chromatin compaction depends on linker length, so different NRL tetra-nucleosomes would explain the different compaction patterns [60]. The authors observed that all four NRLs exhibited the same zigzag patterns, but H1 bound differently according to the DNA linker length [60]. The short NRL resulted in the eviction of H1 binding, which promotes transcription, in line with previous functionality studies [60,75]. H1 positioning in the dyad was shifted slightly depending on DNA length [60]. As expected with the exclusion of salt, the authors [60] did not observe a different twisting pattern, as seen in Takizawa et al. [53], with MgCl_2_. The different arrays show a significant difference between them and make possible the analysis of functional aspects of chromatin, such as linker length.

The importance of array structures is highlighted when we compare the structure of H1-bound mono-, di-, and tetra-nucleosomes (Figure 2). The structures have similar DNA composition based on the synthetic sequence W601. However, the di-nucleosome was reconstituted on sticky-ended DNA, while the mono- and tetra-nucleosomes were reconstituted on blunt-ended DNA. All three structures have human histones, with the histones H3 and H2B having small sequence variations in the histone core. The linker histone H1 used in the di-nucleosome is H1.x, which differs from the mono- and tetra-nucleosome that used H1.4. Despite similar DNA sequences and histone composition, the arrays showcase significant differences in the DNA pathways. The arrays display similar DNA entry patterns; however, DNA exit paths differ significantly even with short DNA linkers (Figure 2). None of the arrays have visible differences in the histone core, which would explain the lack of changes observed in the mono-nucleosome structures. This emphasizes the contribution of arrays to understanding chromatin folding, as this DNA pathway heterogeneity likely mimics higher-order chromatin folding [56,57].

### The role of arrays in the study of chromatin binding partners

Most mono-nucleosome structures in complex with binding partners exhibit no change to the nucleosome core structure (Table 1) [12,14,17]. Although the mono-nucleosome has been pivotal in determining binding sites, essential residues, and stoichiometry, the nucleosome structures are visually similar and provide no information on their possible structural effects on chromatin. Array complexes can emphasize the role of partners on adjacent nucleosomes and their overall impact on chromatin folding dynamics. We will focus on three examples highlighting the need for arrays, including two remodelers and a PTM writer. Thus far, our understanding is limited by interactions only with the mono-nucleosome.

Some complexes, such as ISW1a and embryonic stem cell Brahma-associate factor complex (esBAF), are predicted to have the di-nucleosome as a binding substrate [67,76]. Chromatin remodeling can result from modification by ATP-dependent remodeling complexes, which can slide nucleosomes to expose or hide DNA [77]. Such changes are not visible in the mono-nucleosome, as this model does not allow for nucleosome sliding due to DNA length restrictions. The ISW1a remodeler, when bound to the di-nucleosome, has provided critical insights into its mechanism. Previously described as a ‘gene ruler’, ISW1a’s interaction with the di-nucleosome revealed new details. The loc3 subunit senses the NCP2 of the nucleosome array rather than the linker DNA. Additionally, the NegC subunit plays a key role in regulating the remodeler’s activity through an auto-inhibitory mechanism. When NegC unfolds, it activates the motor subunit, but as the DNA linker length shortens, NegC folds back and inhibits motor activity, thereby controlling the remodeler’s function [67]. Another well-known remodeler is the SWI/SNF complex [78-80]. The 12-member SWI/SNF complex structure was solved with the mono-nucleosome [19]. The crystal structure confirmed that the subcomplex Snf6 directly binds to the nucleosomal DNA at the exit point, whereas no changes were observed in the nucleosome structure. Although the structure has not been solved, the previous work [66] suggests that SWI/SNF would be a candidate for remodeling of a di-nucleosome. This may reveal detailed changes in the histone–DNA interactions caused by the remodeler.

The gene-activation remodeler, Chd1, is known to partially unwrap DNA from ubiquitinylated nucleosomes [14]. In this case, the NCP shows little change despite linker lengths protruding from the core. From a multi-nucleosome perspective, Chd1 binding to a hexasome–nucleosome complex provided key insight into the remodeler’s mechanism of action [81]. The cryo-EM structure of this hexasome–nucleosome array bound to Chd1 revealed that the ATPase domain of Chd1 interacts with superhelical location (SHL) 2 of the hexasome, pushing the hexasome further away from the nucleosome. In a second structure, the H2A–H2B dimer was reinstalled by FACT, the newly incorporated nucleosome caused the DNA binding domain of Chd1 to shift from the ATPase domain, suggesting that the nucleosome had returned to its original position [81]. This remodeler complex’s large size (200 kDa) could also promote steric effects when looking at a tri-nucleosome array, for example. Another complex that recently gained attention as a cancer therapy, targets the ALC1 remodeler, recruited upon DNA damage detection [12,82]. The inactivation of ALC1 induces the death of recombination-deficient cancer cells, and its function has been associated with nucleosome sliding [83]. Cryo-EM and crystallography data of the ALC1 reveal that the ATPase lobe of this complex binds at the H4 tail with little to no change in the NCP [12,13]. Although challenging, there is a need for structural dynamic studies of nucleosome sliding by remodelers that could be accomplished by a larger nucleosome array. Using a tetra-nucleosome with a longer DNA linker length could facilitate analysis of nucleosome sliding as the space between two nucleosomes would be expected to change.

PTMs, such as acetylation and methylation, are dependent on transferases. Methyltransferase SUV420H1 catalyzes a host of lysine methylation events [84]; for example, it transfers two methyl groups onto H4 lysine residue 20 (H4K20me2) [24]. A recently solved structure of SUV420H1 in complex with a mono-nucleosome showed that the enzyme preferentially recognizes H2A.Z containing nucleosomes [24]. Knowing that the catalytic domain of the methyltransferase interacts with the histone H4 tail and nucleosomal DNA, it would be expected to cause changes in the histones and DNA of an array [13]. Like most methyltransferases, the arginine-rich motif of SUV420H1 binds to the acidic patch of the H2A–H2B dimer in the nucleosomes [85]. The authors hypothesize that methylation could spread to neighboring or non-adjacent canonical nucleosomes [24]. Similar effects have been observed for the polycomb repressive complex 2 (PRC2) complex, a gene silencer that methylates lysine 27 of histone H3. Previous studies demonstrated that PRC2 exhibits higher activity on di-nucleosomes [86], and cryo-EM images of a di-nucleosome bound to the PRC2 provide insight into the mechanism of methylation spreading [87]. The di-nucleosome, which contains one trimethylated lysine nucleosome and one unmodified nucleosome, revealed that PRC2 interacts with both nucleosomes, even at varying linker lengths. The complex binds to the H3 tail and nucleosomal DNA but does not interact with the histone core acidic patch or the linker DNA. These findings not only deepen our understanding of the PRC2-mediated methylation process but also provide a foundation for extending these studies to other chromatin-modifying complexes. The observed interactions between chromatin binding partners and nucleosome arrays offer a valuable perspective that could be applied to broader investigations into gene regulation, chromatin remodeling, and epigenetic mechanisms across various systems.

### In-solution techniques for the analysis of nucleosome dynamics

The mono-nucleosome as a model system to understand chromatin has been used in various in-solution approaches, including nuclear magnetic resonance (NMR) [69,88-92], Förster resonance energy transfer (FRET) [93,94], and multiple types of single-molecule studies. Single-molecule approaches include detection techniques (e.g. smFRET-based approaches [95-97]) and manipulation techniques (e.g. optical tweezers, magnetic force, atomic force microscopy (AFM), and electron force microscopy [96,98-100]). For instance, time-lapse AFM studies showed that CENPA octamers rapidly transfer to different DNA substrates [98]. Multiple FRET studies have analyzed the effects of acidic patch-binding peptides on the structure of nucleosomes and revealed that the structure compacts upon binding [101-103]. Hydrogen–deuterium exchange mass spectrometry (HDX-MS) was used to gather evidence of H4 tail interactions [104]. The H4 tail showed a high degree of solvent exposure in the tetramer but negligible exposure in the nucleosome context [104]. This indicated that H4 could be interacting with the nucleosome surface. In-solution techniques continue to provide insight into dynamic changes and conformations of the mono-nucleosome, which are challenging to analyze in techniques that provide data about static views of the nucleosome. New technologies have allowed the growth of these approaches to extend to multi-nucleosome systems. Contrast variation SAXS allowed the monitoring of nucleic acids only, with no interference from protein diffraction signals. The study showed two different populations of tri-nucleosomes in solution. One population had the NCP1 and NCP3 stacked together, while the other had no interaction between the flanking nucleosomes [73].

SAXS has proven helpful in analyzing multi-nucleosome arrays in solution [73]. Based on their application to the mono-nucleosome, promising approaches for studying multi-nucleosome dynamics include but are not limited to FRET [93,95,105], HDX-MS [104,106-108], and NMR [90,91]. NMR size limitations may cause a challenge in the analysis of multi-nucleosome arrays, but its capabilities continue to advance [109,110]. FRET analyzed biomolecule dynamics and detected different nucleosomes during assembly and disassembly [105]. This technique has the potential to study high-resolution folding and unfolding patterns of nucleosome arrays. HDX-MS is a high throughput, in-solution technique that monitors the structural and dynamic features of proteins [108,111,112]. This technique is viable, requiring low sample consumption, achieving sub-peptide level resolution, and detecting changes in highly disordered regions such as histone tails [104,106-108]. The HDX-MS analysis of nucleosome arrays could significantly increase our understanding of the histone interactions in chromatin. However, technical challenges, such as protein concentrations, proper proteolysis, low signal-to-noise ratios, and appropriate fluorophores, must be addressed for both techniques [113,114]. The applications of in-solution methods to multi-nucleosome arrays are still in their beginning stages [95,105,115], but based on the growing evidence, they will be a valuable tool for future chromatin studies.

**Perspectives**

- The mono-nucleosome is an important model for better understanding chromatin mechanisms. It informs on stoichiometry, active sites, relevant residues, and localized effects on the nucleosome but not in the chromatin context.
- Multi-nucleosome arrays provide a more chromatin-like environment.
- Combined with in-solution techniques, such as HDX-MS, FRET, NMR, and SAXS, we can investigate chromatin folding, binding partner incorporation, and chromatin remodeling effects.

## Open access statement

This article has been published open access under our Subscribe to Open programme, made possible through the support of our subscribing institutions, learn more here: https://portlandpress.com/pages/open_access_options_and_prices#conditional

## Abbreviations

AFM: atomic force microscopy
CENP-A: Centromeric protein A
FRET: Förster resonance energy transfer
HDX-MS: hydrogen-deuterium exchange mass spectrometry
ISWI: Imitation switch
NCP: nucleosome core particle
NMR: nuclear magnetic resonance
NRL: nucleosome repeat length
PRC2: Polycomb repressive complex 2
PTM: post-translation modification
SAXS: small-angle x-ray scattering
SHL: superhelical location
SWI/SNF: switch/sucrose non-fermentable
W601: Widom 601
bp: base pair

## References

[1] Luger K., Mäder A.W., Richmond R.K., Sargent D.F., Richmond T.J (1997). Crystal structure of the nucleosome core particle at 2.8 A resolution. Nat. New Biol..

[2] Hergeth S.P., Schneider R (2015). The H1 linker histones: multifunctional proteins beyond the nucleosomal core particle. EMBO Rep..

[3] Wang M., Li J., Wang Y., Fu H., Qiu H., Li Y. (2023). Single-molecule study reveals Hmo1, not Hho1, promotes chromatin assembly in budding yeast. MBio.

[4] Szerlong H.J., Hansen J.C (2011). Nucleosome distribution and linker DNA: connecting nuclear function to dynamic chromatin structure. Biochem. Cell Biol..

[5] Correll S.J., Schubert M.H., Grigoryev S.A (2012). Short nucleosome repeats impose rotational modulations on chromatin fibre folding. EMBO J..

[6] Valouev A., Johnson S.M., Boyd S.D., Smith C.L., Fire A.Z., Sidow A (2011). Determinants of nucleosome organization in primary human cells. Nat. New Biol..

[7] Ghoneim M., Fuchs H.A., Musselman C.A (2021). Histone tail conformations: a fuzzy affair with DNA. Trends Biochem. Sci..

[8] Brouwer T., Pham C., Kaczmarczyk A., De Voogd W.-J., Botto M., Vizjak P. (2021). A critical role for linker DNA in higher-order folding of chromatin fibers. Nucleic Acids Res..

[9] Kobayashi W., Kurumizaka H (2019). Structural transition of the nucleosome during chromatin remodeling and transcription. Curr. Opin. Struct. Biol..

[10] Kenzaki H., Takada S (2021). Linker DNA length is a key to tri-nucleosome folding. J. Mol. Biol..

[11] Liu C.-P., Yu Z., Xiong J., Hu J., Song A., Ding D. (2023). Structural insights into histone binding and nucleosome assembly by chromatin assembly factor-1. Science.

[12] Bacic L., Gaullier G., Sabantsev A., Lehmann L.C., Brackmann K., Dimakou D. (2021). Structure and dynamics of the chromatin remodeler ALC1 bound to a PARylated nucleosome. Elife.

[13] Wang L., Chen K., Chen Z (2021). Structural basis of ALC1/CHD1L autoinhibition and the mechanism of activation by the nucleosome. Nat. Commun..

[14] Nodelman I.M., Das S., Faustino A.M., Fried S.D., Bowman G.D., Armache J.P (2022). Nucleosome recognition and DNA distortion by the Chd1 remodeler in a nucleotide-free state. Nat. Struct. Mol. Biol..

[15] Farnung L., Ochmann M., Cramer P (2020). Nucleosome-CHD4 chromatin remodeler structure maps human disease mutations. Elife.

[16] Lee S.C., Adams D.W., Ipsaro J.J., Cahn J., Lynn J., Kim H.-S (2023). Chromatin remodeling of histone H3 variants by DDM1 underlies epigenetic inheritance of DNA methylation. Cell.

[17] Kunert F., Metzner F.J., Jung J., Höpfler M., Woike S., Schall K. (2022). Structural mechanism of extranucleosomal DNA readout by the INO80 complex. Sci. Adv..

[18] Wu H., Muñoz E.N., Hsieh L.J., Chio U.S., Gourdet M.A., Narlikar G.J. (2023). Reorientation of INO80 on hexasomes reveals basis for mechanistic versatility. Science.

[19] He Z., Chen K., Ye Y., Chen Z (2021). Structure of the SWI/SNF complex bound to the nucleosome and insights into the functional modularity. Cell Discov..

[20] Baker R.W., Reimer J.M., Carman P.J., Turegun B., Arakawa T., Dominguez R. (2021). Structural insights into assembly and function of the RSC chromatin remodeling complex. Nat. Struct. Mol. Biol..

[21] Yuan J., Chen K., Zhang W., Chen Z (2022). Structure of human chromatin-remodelling PBAF complex bound to a nucleosome. Nat. New Biol..

[22] Farnung L., Ochmann M., Engeholm M., Cramer P (2021). Structural basis of nucleosome transcription mediated by Chd1 and FACT. Nat. Struct. Mol. Biol..

[23] Liu Y., Zhang Y., Xue H., Cao M., Bai G., Mu Z. (2021). Cryo-EM structure of SETD2/Set2 methyltransferase bound to a nucleosome containing oncohistone mutations. Cell Discov..

[24] Huang L., Wang Y., Long H., Zhu H., Wen Z., Zhang L (2023). Structural insight into H4K20 methylation on H2A.Z-nucleosome by SUV420H1. Mol. Cell..

[25] Dong S., Li H., Wang M., Rasheed N., Zou B., Gao X. (2023). Structural basis of nucleosome deacetylation and DNA linker tightening by Rpd3S histone deacetylase complex. Cell Res..

[26] Guan R., Lian T., Zhou B.-R., He E., Wu C., Singleton M. (2021). Structural and dynamic mechanisms of CBF3-guided centromeric nucleosome formation. Nat. Commun..

[27] Wang H., Xiong L., Cramer P (2021). Structures and implications of TBP-nucleosome complexes. Proc. Natl. Acad. Sci. U.S.A..

[28] Guan R., Lian T., Zhou B.R., Wheeler D., Bai Y (2023). Structural mechanism of LIN28B nucleosome targeting by OCT4. Mol. Cell..

[29] Das S.K., Huynh M.T., Lee T.H (2023). Spontaneous histone exchange between nucleosomes. J. Biol. Chem..

[30] Armeev G.A., Kniazeva A.S., Komarova G.A., Kirpichnikov M.P., Shaytan A.K (2021). Histone dynamics mediate DNA unwrapping and sliding in nucleosomes. Nat. Commun..

[31] Marunde M.R., Fuchs H.A., Burg J.M., Popova I.K., Vaidya A., Hall N.W. (2024). Nucleosome conformation dictates the histone code. Elife.

[32] Weinzapfel E.N., Fedder-Semmes K.N., Sun Z.W., Keogh M.C (2024). Beyond the tail: the consequence of context in histone post-translational modification and chromatin research. Biochem. J..

[33] Villaseñor R., Baubec T (2021). Regulatory mechanisms governing chromatin organization and function. Curr. Opin. Cell Biol..

[34] Li S., Wei T., Panchenko A.R (2023). Histone variant H2A.Z modulates nucleosome dynamics to promote DNA accessibility. Nat. Commun..

[35] Talbert P.B., Henikoff S (2021). Histone variants at a glance. J. Cell. Sci..

[36] Zhao H., Guo M., Zhang F., Shao X., Liu G., Xing Y. (2021). Nucleosome assembly and disassembly in vitro are governed by chemical kinetic principles. Front. Cell Dev. Biol..

[37] Travers A., Hiriart E., Churcher M., Caserta M., Di Mauro E (2010). The DNA sequence-dependence of nucleosome positioning in vivo and in vitro. J. Biomol. Struct. Dyn..

[38] Jain K., Marunde M.R., Burg J.M., Gloor S.L., Joseph F.M., Poncha K.F. (2023). An acetylation-mediated chromatin switch governs H3K4 methylation read-write capability. Elife.

[39] Maeshima K., Ide S., Babokhov M (2019). Dynamic chromatin organization without the 30-nm fiber. Curr. Opin. Cell Biol..

[40] Collepardo-Guevara R., Schlick T (2014). Chromatin fiber polymorphism triggered by variations of DNA linker lengths. Proc. Natl. Acad. Sci. U.S.A..

[41] Li Y., Zhang H., Li X., Wu W., Zhu P (2023). Cryo-ET study from in vitro to in vivo revealed a general folding mode of chromatin with two-start helical architecture. Cell Rep..

[42] Maeshima K., Iida S., Tamura S (2021). Physical nature of chromatin in the nucleus. Cold Spring Harb. Perspect. Biol..

[43] Murphy K.J., Cutter A.R., Fang H., Postnikov Y.V., Bustin M., Hayes J.J (2017). HMGN1 and 2 remodel core and linker histone tail domains within chromatin. Nucleic Acids Res..

[44] Ohtomo H., Ito S., McKenzie N.J., Uckelmann M., Wakamori M., Ehara H (2023). H2A ubiquitination alters H3-tail dynamics on linker-DNA to enhance H3K27 methylation. J. Mol. Biol..

[45] Luque A., Ozer G., Schlick T (2016). Correlation among DNA linker length, linker histone concentration, and histone tails in chromatin. Biophys. J..

[46] Lowary P.T., Widom J (1998). New DNA sequence rules for high affinity binding to histone octamer and sequence-directed nucleosome positioning. J. Mol. Biol..

[47] Wang H., Farnung L., Dienemann C., Cramer P (2020). Structure of H3K36-methylated nucleosome-PWWP complex reveals multivalent cross-gyre binding. Nat. Struct. Mol. Biol..

[48] Horikoshi N., Arimura Y., Taguchi H., Kurumizaka H (2016). Crystal structures of heterotypic nucleosomes containing histones H2A.Z and H2A. Open Biol..

[49] Kohestani H., Wereszczynski J (2021). Effects of H2A.B incorporation on nucleosome structures and dynamics. Biophys. J..

[50] Hermans N., Huisman J.J., Brouwer T.B., Schächner C., van Heusden G.P.H., Griesenbeck J (2017). Toehold-enhanced LNA probes for selective pull down and single-molecule analysis of native chromatin. Sci. Rep..

[51] Müller O., Kepper N., Schöpflin R., Ettig R., Rippe K., Wedemann G (2014). Changing chromatin fiber conformation by nucleosome repositioning. Biophys. J..

[52] Bowman G.D., Poirier M.G (2015). Post-translational modifications of histones that influence nucleosome dynamics. Chem. Rev..

[53] Takizawa Y., Ho C.-H., Tachiwana H., Matsunami H., Kobayashi W., Suzuki M. (2020). Cryo-EM structures of centromeric tri-nucleosomes containing a central CENP-A nucleosome. Structure.

[54] Morgan A., LeGresley S., Fischer C (2020). Remodeler catalyzed nucleosome repositioning: Influence of structure and stability. Int. J. Mol. Sci..

[55] Roulland Y., Ouararhni K., Naidenov M., Ramos L., Shuaib M., Syed S.H (2016). The flexible ends of CENP-A nucleosome are required for mitotic fidelity. Mol. Cell..

[56] Maeshima K., Rogge R., Tamura S., Joti Y., Hikima T., Szerlong H. (2016). Nucleosomal arrays self-assemble into supramolecular globular structures lacking 30-nm fibers. EMBO J..

[57] Rogge R.A., Hansen J.C (2015). Sedimentation velocity analysis of large oligomeric chromatin complexes using interference detection. Meth. Enzymol..

[58] Mishra L.N., Hayes J.J (2018). A nucleosome-free region locally abrogates histone H1-dependent restriction of linker DNA accessibility in chromatin. J. Biol. Chem..

[59] Soman A., Wong S.Y., Korolev N., Surya W., Lattmann S., Vogirala V.K. (2022). Columnar structure of human telomeric chromatin. Nat. New Biol..

[60] Dombrowski M., Engeholm M., Dienemann C., Dodonova S., Cramer P (2022). Histone H1 binding to nucleosome arrays depends on linker DNA length and trajectory. Nat. Struct. Mol. Biol..

[61] Li W., Chen P., Yu J., Dong L., Liang D., Feng J (2016). Fact remodels the tetranucleosomal unit of chromatin fibers for gene transcription. Mol. Cell..

[62] Adhireksan Z., Sharma D., Lee P.L., Bao Q., Padavattan S., Shum W.K. (2021). Engineering nucleosomes for generating diverse chromatin assemblies. Nucleic Acids Res..

[63] Adhireksan Z., Sharma D., Lee P.L., Davey C.A (2020). Near-atomic resolution structures of interdigitated nucleosome fibres. Nat. Commun..

[64] Schalch T., Duda S., Sargent D.F., Richmond T.J (2005). X-ray structure of a tetranucleosome and its implications for the chromatin fibre. Nat. New Biol..

[65] Garcia-Saez I., Menoni H., Boopathi R., Shukla M.S., Soueidan L., Noirclerc-Savoye M (2018). Structure of an H1-bound 6-nucleosome array reveals an untwisted two-start chromatin fiber conformation. Mol. Cell..

[66] Kato D., Osakabe A., Arimura Y., Mizukami Y., Horikoshi N., Saikusa K. (2017). Crystal structure of the overlapping dinucleosome composed of hexasome and octasome. Science.

[67] Li L., Chen K., Sia Y., Hu P., Ye Y., Chen Z (2024). Structure of the ISW1a complex bound to the dinucleosome. Nat. Struct. Mol. Biol..

[68] Ekundayo B., Richmond T.J., Schalch T (2017). Capturing structural heterogeneity in chromatin fibers. J. Mol. Biol..

[69] Zhou B.-R., Feng H., Kale S., Fox T., Khant H., de Val N (2021). Distinct Structures and Dynamics of Chromatosomes with Different Human Linker Histone Isoforms. Mol. Cell..

[70] Sharma D., De Falco L., Padavattan S., Rao C., Geifman-Shochat S., Liu C.-F. (2019). PARP1 exhibits enhanced association and catalytic efficiency with γH2A.X-nucleosome. Nat. Commun..

[71] Ghoneim M., Musselman C.A (2023). Protocol to prepare doubly labeled fluorescent nucleosomes for single-molecule fluorescence microscopy. STAR Protoc..

[72] Schwarz P.M., Felthauser A., Fletcher T.M., Hansen J.C (1996). Reversible oligonucleosome self-association: dependence on divalent cations and core histone tail domains. Biochemistry.

[73] Mauney A.W., Muthurajan U.M., Luger K., Pollack L (2021). Solution structure(s) of trinucleosomes from contrast variation SAXS. Nucleic Acids Res..

[74] Soman A., Liew C.W., Teo H.L., Berezhnoy N.V., Olieric V., Korolev N. (2020). The human telomeric nucleosome displays distinct structural and dynamic properties. Nucleic Acids Res..

[75] Fyodorov D.V., Zhou B.R., Skoultchi A.I., Bai Y (2018). Emerging roles of linker histones in regulating chromatin structure and function. Nat. Rev. Mol. Cell Biol..

[76] Klein D.C., Troy K., Tripplehorn S.A., Hainer S.J (2023). The esBAF and ISWI nucleosome remodeling complexes influence occupancy of overlapping dinucleosomes and fragile nucleosomes in murine embryonic stem cells. BMC Genomics.

[77] Clapier C.R., Iwasa J., Cairns B.R., Peterson C.L (2017). Mechanisms of action and regulation of ATP-dependent chromatin-remodelling complexes. Nat. Rev. Mol. Cell Biol..

[78] Bhoite L.T., Yu Y., Stillman D.J (2001). The Swi5 activator recruits the Mediator complex to the HO promoter without RNA polymerase II. Genes Dev..

[79] Wang L., Tang J (2023). SWI/SNF complexes and cancers. Gene.

[80] Chen K., Yuan J., Sia Y., Chen Z (2023). Mechanism of action of the SWI/SNF family complexes. Nucleus.

[81] Engeholm M., Roske J.J., Oberbeckmann E., Dienemann C., Lidschreiber M., Cramer P (2024). Resolution of transcription-induced hexasome-nucleosome complexes by Chd1 and FACT. Mol. Cell..

[82] Hewitt G., Borel V., Segura-Bayona S., Takaki T., Ruis P., Bellelli R (2021). Defective ALC1 nucleosome remodeling confers PARPi sensitization and synthetic lethality with HRD. Mol. Cell..

[83] Verma P., Zhou Y., Cao Z., Deraska P.V., Deb M., Arai E. (2021). ALC1 links chromatin accessibility to PARP inhibitor response in homologous recombination-deficient cells. Nat. Cell Biol..

[84] Kato H., Hayami S., Ueno M., Suzaki N., Nakamura M., Yoshimura T. (2024). Histone methyltransferase SUV420H1/KMT5B contributes to poor prognosis in hepatocellular carcinoma. Cancer Sci..

[85] Lehmann L.C., Bacic L., Hewitt G., Brackmann K., Sabantsev A., Gaullier G. (2020). Mechanistic insights into regulation of the ALC1 remodeler by the nucleosome acidic patch. Cell Rep..

[86] Martin C., Cao R., Zhang Y (2006). Substrate preferences of the EZH2 histone methyltransferase complex. J. Biol. Chem..

[87] Poepsel S., Kasinath V., Nogales E (2018). Cryo-EM structures of PRC2 simultaneously engaged with two functionally distinct nucleosomes. Nat. Struct. Mol. Biol..

[88] Zhou B.-R., Feng H., Kato H., Dai L., Yang Y., Zhou Y. (2013). Structural insights into the histone H1-nucleosome complex. Proc. Natl. Acad. Sci. U.S.A..

[89] Grigoryev S.A (2018). Chromatin Higher-Order Folding: A Perspective with Linker DNA Angles. Biophys. J..

[90] Ackermann B.E., Debelouchina G.T (2021). Emerging contributions of solid-state NMR spectroscopy to chromatin Structural biology. Front. Mol. Biosci..

[91] Shi X., Prasanna C., Nagashima T., Yamazaki T., Pervushin K., Nordenskiöld L (2018). Structure and dynamics in the nucleosome revealed by solid-state NMR. Angew. Chem. Int. Ed. Engl..

[92] Zandian M., Gonzalez Salguero N., Shannon M.D., Purusottam R.N., Theint T., Poirier M.G. (2021). Conformational dynamics of histone H3 tails in chromatin. J. Phys. Chem. Lett..

[93] Gansen A., Hieb A.R., Böhm V., Tóth K., Langowski J (2013). Closing the gap between single molecule and bulk FRET analysis of nucleosomes. PLoS ONE.

[94] Das S.K., Huynh M.T., Gao J., Sengupta B., Yadav S.P., Lee T.H (2023). Methods to investigate nucleosome structure and dynamics with single-molecule FRET. Methods.

[95] Kilic S., Felekyan S., Doroshenko O., Boichenko I., Dimura M., Vardanyan H (2018). Single-molecule FRET reveals multiscale chromatin dynamics modulated by HP1α. Nat. Commun..

[96] Sivkina A.L., Karlova M.G., Valieva M.E., McCullough L.L., Formosa T., Shaytan A.K (2022). Electron microscopy analysis of ATP-independent nucleosome unfolding by FACT. Commun. Biol..

[97] Mimitou E.P., Lareau C.A., Chen K.Y., Zorzetto-Fernandes A.L., Hao Y., Takeshima Y. (2021). Scalable, multimodal profiling of chromatin accessibility, gene expression and protein levels in single cells. Nat. Biotechnol..

[98] Stumme-Diers M.P., Stormberg T., Sun Z., Lyubchenko Y.L (2019). Probing the structure and dynamics of nucleosomes using atomic force microscopy imaging. J. Vis. Exp..

[99] Bustamante C.J., Chemla Y.R., Liu S., Wang M.D (2021). Optical tweezers in single-molecule biophysics. Nat. Rev. Methods Primers.

[100] Chien F.-T., van Heijden T (2014). Characterization of nucleosome unwrapping within chromatin fibers using magnetic tweezers. Biophys. J..

[101] Liu Z., Wu Y., Mao X., Kwan K.C.J., Cheng X., Li X. (2023). Development of multifunctional synthetic nucleosomes to interrogate chromatin-mediated protein interactions. Sci. Adv..

[102] Oleinikov P.D., Fedulova A.S., Armeev G.A., Motorin N.A., Singh-Palchevskaia L., Sivkina A.L. (2023). Interactions of Nucleosomes with Acidic Patch-Binding Peptides: A Combined Structural Bioinformatics, Molecular Modeling, Fluorescence Polarization, and Single-Molecule FRET Study. Int. J. Mol. Sci..

[103] Fang J., Nevin P., Kairys V., Venclovas Č., Engen J.R., Beuning P.J (2014). Conformational analysis of processivity clamps in solution demonstrates that tertiary structure does not correlate with protein dynamics. Structure.

[104] Karch K.R., Coradin M., Zandarashvili L., Kan Z.-Y., Gerace M., Englander S.W (2018). Hydrogen-deuterium exchange coupled to top- and middle-down mass spectrometry reveals histone tail dynamics before and after nucleosome assembly. Structure.

[105] Gansen A., Valeri A., Hauger F., Felekyan S., Kalinin S., Tóth K. (2009). Nucleosome disassembly intermediates characterized by single-molecule FRET. Proc. Natl. Acad. Sci. U.S.A..

[106] Bhat Y. A., Bhat J. Y., Amin S., Udgaonkar J. B., Wani A. H (2023). HDX-MS reveals concealed conformations of ISWI during different stages of nucleosome sliding. bioRxiv.

[107] Liu W.H., Zheng J., Feldman J.L., Klein M.A., Kuznetsov V.I., Peterson C.L (2020). Multivalent interactions drive nucleosome binding and efficient chromatin deacetylation by SIRT6. Nat. Commun..

[108] Papanastasiou M., Mullahoo J., DeRuff K.C., Bajrami B., Karageorgos I., Johnston S.E (2019). Chasing tails: cathepsin-L improves structural analysis of histones by HX-MS. Mol. Cell. Proteomics..

[109] Rosenzweig R., Kay L.E (2014). Bringing dynamic molecular machines into focus by methyl-TROSY NMR. Annu. Rev. Biochem..

[110] Musselman C.A., Kutateladze T.G (2022). Visualizing Conformational Ensembles of the Nucleosome by NMR. ACS Chem. Biol..

[111] Narang D., Lento C., J Wilson D (2020). HDX-MS: an analytical tool to capture protein motion in action. Biomedicines.

[112] Filandrova R., Kavan D., Kadek A., Novak P., Man P (2021). Studying Protein-DNA Interactions by Hydrogen/Deuterium Exchange Mass Spectrometry. Methods Mol. Biol..

[113] Masson G.R., Burke J.E., Ahn N.G., Anand G.S., Borchers C., Brier S. (2019). Recommendations for performing, interpreting and reporting hydrogen deuterium exchange mass spectrometry (HDX-MS) experiments. Nat. Methods.

[114] Frueh D.P., Goodrich A.C., Mishra S.H., Nichols S.R (2013). NMR methods for structural studies of large monomeric and multimeric proteins. Curr. Opin. Struct. Biol..

[115] Poirier M.G., Oh E., Tims H.S., Widom J (2009). Dynamics and function of compact nucleosome arrays. Nat. Struct. Mol. Biol..

